# Neuroprotective Activity of Lavender Oil on Transient Focal Cerebral Ischemia in Mice

**DOI:** 10.3390/molecules17089803

**Published:** 2012-08-15

**Authors:** Dong Wang, Xuan Yuan, Ting Liu, Liangliang Liu, Yanli Hu, Zhenhua Wang, Qiusheng Zheng

**Affiliations:** 1 Qianfoshan Hospital of Shandong Province, Jinan 250005, China; Email: wangdong9859@sina.com.cn; 2 Lanzhou University Second Hospital, Lanzhou 730000, China; Email: xyuan2011@lzu.edu.cn; 3 Key Laboratory of Xinjiang Endemic Phytomedicine Resources Ministry of Education, School of Pharmacy, Shihezi University, Shihezi 832002, China; Email: liuting0908@126.com (T.L.); lll80231226@163.com (L.L.); wzx330@163.com (Y.H.); 4 School of life Sciences, Yantai University, Yantai 264005, China

**Keywords:** lavender oil, cerebral ischemia/reperfusion, oxidative damage；ROS, antioxidant

## Abstract

The air-dried aerial parts of *Lavandula angustifolia* Mill, a traditional Uygur herbal drug, is used as resuscitation-inducing therapy to treat neurodisfunctions, such as stroke. This study was designed to assess the neuroprotective effects of lavender oil against ischemia/reperfusion (IR) injury in mice. Focal cerebral ischemia was induced by the intraluminal occlusion method with a nylon string. The neurodysfuntion was evaluated by neurological deficit and the infarct area was showed by 2,3,5-triphenyltetrazolium chloride (TTC) staining. The histopathological changes were observed by hematoxylin and eosin staining. The levels of mitochondria-generated reactive oxygen species (ROS), malondialdehyde (MDA) and carbonyl, the ratio of reduced glutathione (GSH)/glutathione disulfide (GSSG), the activities of superoxide dismutase (SOD), catalase (CAT) and glutathion peroxidase (GSH-Px) in brain tissue were measured to estimate the oxidative stress state. Neurological deficit, infarct size, histopathology changes and oxidative stress markers were evaluated after 22 h of reperfusion. In comparison with the model group, treatment with lavender oil significantly decreased neurological deficit scores, infarct size, the levels of MDA, carbonyl and ROS, and attenuated neuronal damage, upregulated SOD, CAT, GSH-Px activities and GSH/GSSG ratio. These results suggested that the neuroprotective effects of lavender oil against cerebral ischemia/reperfusion injury may be attributed to its antioxidant effects.

## 1. Introduction

Cerebral ischemia is one of the leading causes of human death and disability across the World and its incidence is speculated to rise with the increase in the number of the aging population. Preventive treatments intended to reduce the risk factors have been demonstrated beneficial, but there are no efficient treatments except the thrombolytic recombinant tissue plasminogen activator or edaravone [1].

A variety of mechanisms are involved in ischemic brain injury, including excitotoxicity, acidotoxicity, ionic imbalance, peri-infarct depolarization, oxidative and nitrative stress, inflammation and apoptosis [2,3]. Recent studies have demonstrated that the brain damages after ischemia/reperfusion may result from oxidative stress [4,5]. Ischemia/reperfusion leads to an imbalance between antioxidants and accumulations of toxic free radicals increasing the susceptibility of brain tissues to oxidative damage via lipid peroxidation, protein oxidation, DNA oxidation and so on [6]. For this reason, antioxidants have been the focus of studies for developing neuroprotective drugs to be used in cerebral ischemia therapy. Many antioxidants have been investigated in *in vitro* and *in vivo* experiments and some of them have been tested in clinical studies of cerebral ischemia [1,3,7,8,9].

Essential oils, containing a complex mixture of odorous and volatile compounds, are widely used in cosmetics, food industry and household products [10]. Some kind of essential oils exhibited anti-oxidant [11,12], anti-hypoxic-ischemic [13], anti-bacterial and anti-fungal activities [14] muscle relaxant [15]. Lavender essential oil, volatile aromatic oily liquids isolated from *Lavandula* plants by various means, used as folk medicine possesses many therapeutic properties including anti-oxidant [16], antiinflammatory [17], antibacterial [18], anxiolytic and sedative action [19], antiplatelet and antithrombotic activities [20], antimutagenic activity [21], treatment agitation in patients with severe dementia [22]. But the protective effects of lavender oil against cerebral ischemia/reperfusion damage have not been evaluated. Therefore, in the present study, the neuroprotective effects of lavender oil were evaluated in a middle cerebral artery occlusion vehicle group, the underlying neuroprotective mechanisms were also investigated.

## 2. Results and Discussion

### 2.1. Neurological Deficits and Infarct Area

**Figure 1:**
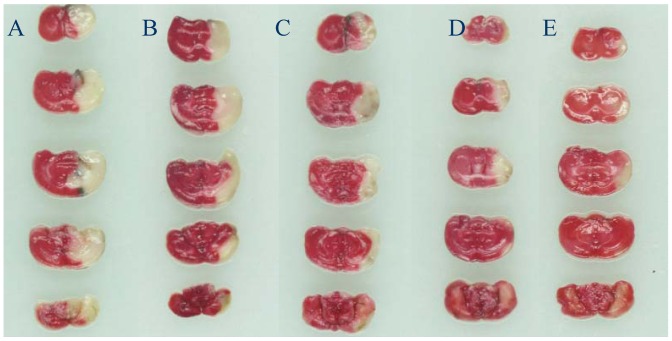
Representative coronal brain sections (1.5 mm thick) after 22 h of reperfusion showing infarction. (**A**) Vehicle group; (**B**) Lavender oil-treated group (50 mg/kg); (**C**) Lavender oil-treated group (100 mg/kg); (**D**) Lavender oil-treated group (200 mg/kg); (**E**) Edaravone-treated group (3 mg/kg).

Figure 1 shows typical photographs of coronal sections of each group. Representative coronal brain sections (1.5 mm thick) stained with 0.5% 2,3,5-triphenyltetrazolium chloride (TTC) after 22 h of reperfusion showing infarction. Red colored region in the TTC-stained sections indicates no-ischemic portion of brain and pale-colored region indicates ischemic portion. The sections were scanned and area of infarction measured using “Photoshop 6.0” image analysis software. The model group had a significantly increased infarct size (pale coloured region) as compared to the sham group. Decreases in infarct size were observed in lavender oil-treated groups in a dose dependent manner (Table 1). The neurological scores after 22 h of reperfusion were given in Table 1. Compared with the sham group, the neurological scores after 22 h of reperfusion increased significantly in the model group (*p* < 0.01). Pretreatment with lavender oil significantly reduced the neurological deficits (*p* < 0.05).

**Table 1:** Effects of lavender oil and edaravone on the neurological deficits and infarct area after 22 h of reperfusion.

Group	Dose (mg/kg)	Neurological deficit scores	Infarct area (%)
Vehicle	/	2.20 ± 0.79	30.14 ± 3.83
Sham	/	0.00 ± 0.00 **	0.00 ± 0.00 **
Lavender oil	50	2.10 ± 0.74	28.11 ± 3.41
Lavender oil	100	2.00 ± 0.82	24.85 ± 2.30 *
Lavender oil	200	1.50 ± 0.71	15.10 ± 2.93 **
Edaravone	3	1.40 ± 0.52 *	11.01 ± 1.42 **

All values given in the table 1 are Mean ± S.E.M.; Differences were considered significant at *p <* 0.05 .* *p <* 0.05,** *p <* 0.01 *vs.* vehicle group.

### 2.2. Histopathology (H&E) Staining

**Figure 2:**
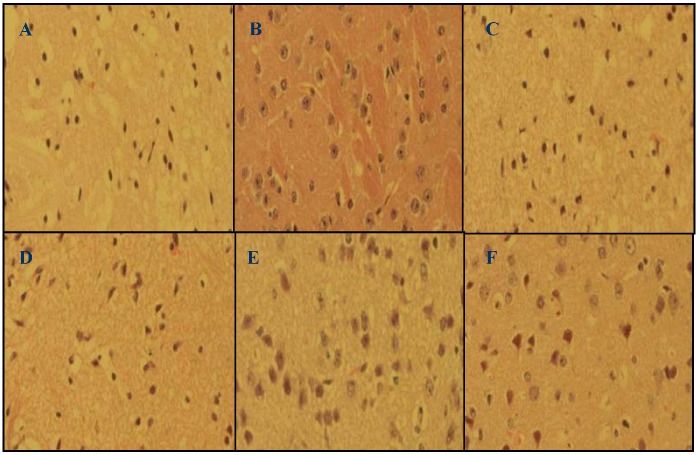
Representative coronal sections stained with hematoxylin-eosin (HE) after 22 h of reperfusion (×400 magniﬁcations). (**A**) Vehicle group; (**B**) Sham group; (**C**) Lavender oil-treated group (50 mg/kg); (**D**) Lavender oil-treated group (100 mg/kg); (**E**) Lavender oil-treated group (200 mg/kg); (**F**) Edaravone-treated group (3 mg/kg).

The typical histopathological architecture of mouse brain was shown in the sham group (Figure 2B). Brains of mice subjected to cerebral ischemia/reperfusion revealed alterations in brain histology in the form of typical colliquative necrosis, reticular softening lesions, nucleus shrinkage or disappearance, cytoplasmic dissolution and cellular edema (Figure 2A). Lavender oil and edaravone markedly reduced these ischemia/reperfusion induced histopathological changes (Figure 2C–F).

### 2.3. Protein Carbonyl Content (PCC) Decreased in Lavender Oil-Treated Groups

**Figure 3:**
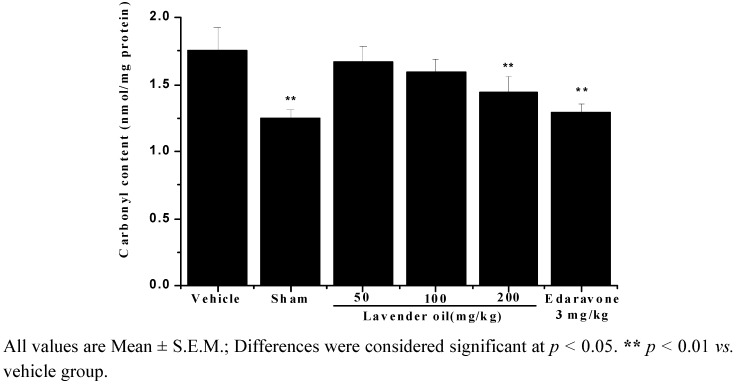
Protein carbonyl content estimated after 22 h of reperfusion.

Protein carbonyl content (PCC) is the most widely used markers of oxidative modification of proteins. It could be demonstrated that oxidative damage to proteins correlates the severity of some diseases, including cerebral ischemia. As shown in Figure 3, the protein carbonyl level increased from basal value of 1.25 ± 0.06 nmol/mg protein in the sham group to 1.75 ± 0.16 nmol/mg protein in the vehicle group. Protein carbonyl formation decreased in the 200 mg/kg lavender oil groups.

### 2.4. Lavender Oil-Treatment Decreased Malondialdehyde (MDA) Content

**Figure 4:**
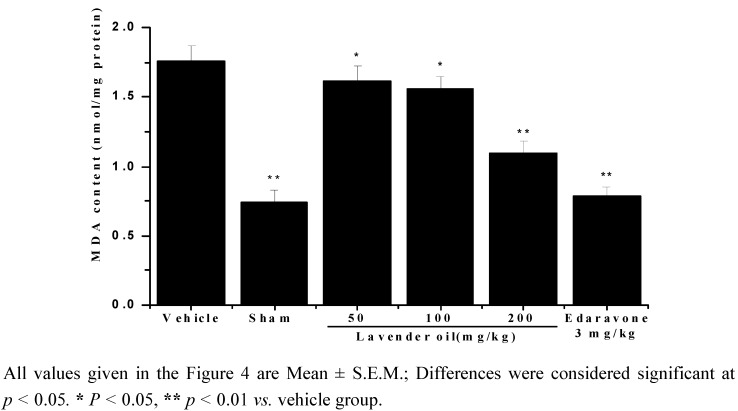
MDA content measured after 22 h of reperfusion.

Malondialdehyde (MDA) formation is widely used as the index of lipid peroxidation. The results clearly demonstrated that ischemia/reperfusion resulted in an increase of MDA formation compared with the sham group and MDA content reduced in a dose dependent manner in the lavender oil groups (Figure 4), *p* < 0.05 at dose of 50 or 100 mg/Kg, and *p* < 0.01 at dose of 200 mg/Kg.

### 2.5. Lavender Oil-Treatment Alleviate the Decrease of Antioxidant Enzyme Activities Induced by IR

As shown in Table 2, cerebral ischemia/reperfusion led to a significant decrease of SOD, CAT and GSH-Px activities. When compared with the vehicle group, lavender oil (200 mg/kg) and edaravone (3 mg/kg) administration greatly elevated these antioxidant enzyme activities.

**Table 2:** Effects of lavender oil and edaravone on the antioxidant enzyme activities after 22 h of reperfusion.

Group	Dose (mg/kg)	CAT (U/mg protein)	SOD (U/mg protein)	GSH-Px (U/mg protein)
Vehicle	/	28.38 ± 10.62	5.90 ± 1.19	27.04 ± 4.24
Sham	/	69.58 ± 13.25 **	12.64 ± 0.78 **	45.13 ± 3.87 **
Lavender oil	50	32.10 ± 8.36	6.22 ± 0.91	29.21 ± 4.15
Lavender oil	100	35.40 ± 5.78	6.73 ± 0.81	33.11 ± 3.77 *
Lavender oil	200	39.92 ± 5.75 *	7.68 ± 0.76 *	34.02 ± 3.51 *
Edaravone	3	56.54 ± 6.50 **	10.76 ± 0.54 **	37.99 ± 3.05 **

All values given in the Table 2 are Mean ± S.E.M.; Differences were considered significant at *p <* 0.05. * p < 0.05, ** p < 0.01 *vs.* vehicle group.

### 2.6. Lavender Oil-Treatment Alleviate the Decrease of GSH/GSSG Ratio Induced by IR

**Figure 5:**
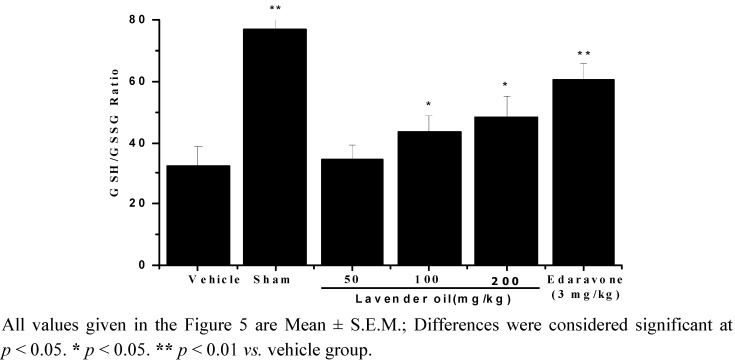
GSH/GSSG ratio measured after 22 h of reperfusion.

GSH/GSSG ratio was shown in Figure 5. Cerebral ischemia/reperfusion led to a significant decrease of GSH/GSSG ratio (*p* < 0.01). The treatments with lavender oil or edaravone increased GSH/GSSG ratio in brain tissue of the cerebral ischemia/reperfusion mice compared with the vehicle group (*p* < 0.05).

### 2.7. Lavender Oil-Treatment Decreased Reactive ROS Formation in Brain Mitochondria

**Figure 6:**
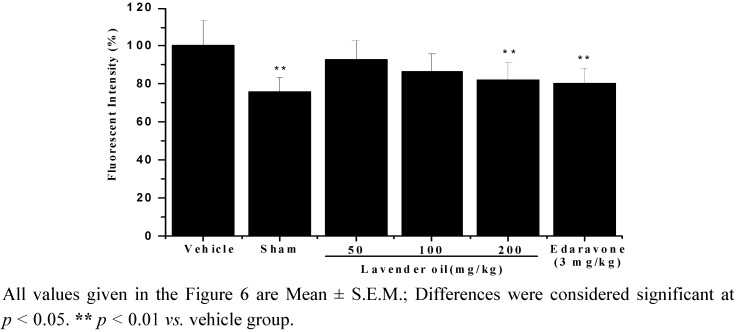
Reactive oxygen species (ROS) estimated after 22 h of reperfusion.

The fluorescent probe 2′,7′-dichlorodihydrofluorescein was used to measure the level of ROS. As shown in Figure 6, the level of ROS in vehicle group was much higher compared with sham group. Administration of lavender oil reduced the ROS level in a concentration-dependent manner compared with the vehicle group (Figure 6).

### 2.8. Discussion

Lavender is reported to be an effective medical plant in treating inflammation, depression, stress and mild anxiety in Europe and the USA, multiple exposures to lavender essential oils could effectively reverse spatial memory deficits induced by dysfunction of the cholinergic system and might provide an opportunity for management neurological abnormalities in dementia conditions [23]. In the present study, we showed an array of evidence that lavender oil exhibited neuroprotective activity against cerebral ischemia/reperfusion damage in a middle cerebral artery occlusion vehicle group. 

The neurological deficits, infarct area and histopathological characterization reveal the neuronal degeneration and necrosis [24]. Neurological deficits were found to be attended by neuronal degeneration and necrosis. The present study showed that the neurological deficit and neuronal degeneration were improved by treatment of lavender oil. The data also revealed that treatment with lavender oil showed a significant reduction in infarct area in ischemia/reperfusion mice. These observations indicated that lavender oil could efficiently prevent the brain injury induced by ischemia/reperfusion.

Edaravone (3-methyl-1-phenyl-2-pyrazolin-5-one), a free radical scavenger, has been used in patients with acute brain infarction since 2001. Edaravone has been shown to prevent brain edema after ischemia and reperfusion injury in animal models and in stroke patients [25]. It is thought that edaravone shows antioxidant actions through enhancement of prostacyclin production, inhibition of lipoxygenase metabolism of arachidonic acid by trapping hydroxyl radicals, inhibition of alloxan-induced lipid peroxidation, and quenching of active oxygen, leading to protection of various cells such as endothelial cells and myocardial cells, against damage by reactive oxygen species (ROS). Side effects of edaravone, including acute renal failure, liver dysfunction, acute allergic reaction, disseminated intravascular coagulation, thrombocytopenia, leukocytopenia and renal dysfunction, during edaravone treatment are occasionally observed by >5%, respectively [25]. Thus, edaravone can be used with a low rate of side effects. No side effects are anticipated if the dose of 30 mg. However, since death due to acute renal failure during edaravone treatment has been reported, this agent is contraindicated for patients with severe renal dysfunction. In addition, edaravone should be carefully used in elderly patients and patients with liver disease, renal disease, hematologic disease, or dehydration.

Lavender oil contains a complex mixture of odorous and volatile compounds, and is widely used in cosmetics, the food industry and household products. Although lavender oil treatment is administered at a relatively higher dose (50–200 mg/Kg) than edaravone, it has little untoward effects, providing a new optional auxiliary therapeutic medicine for the preventing the brain injury induced by ischemia/reperfusion. Another fact should also cause our attention that air-exposed lavender oil can be an important source of exposure to allergenic hydroperoxides [26]. A recent study has suggested that the mutagenic activity of lavender oil can be related to the presence of linalyl acetate, which seems to have an aneugenic agent profile [27].

Protein oxidative damage is inherent to aerobic life. Amino acids of proteins modified by free radicals led to loss of protein structure and function which can occur through denaturation, fragmentation and aggregation. Once oxidized, proteins are degraded by the proteosome complex or lysosomal hydrolases and they can be repaired by antioxidants [28]. Carbonyl content is one of typical biomarkers for determining protein oxidative damage. In the present study, we found that protein carbonyl formation in cerebral ischemia/reperfusion mouse brain increased as compared to sham-operated mice and for the first time showed that the elevated level of carbonyl in brain was decreased by treatment with lavender oil.

It has been shown that the production of free radicals such as superoxide radicals, hydroxyl radicals and hydrogen peroxide are accumulated after cerebral ischemia/reperfusion. And the rates of oxidative metabolic activities and the levels of polyunsaturated fatty acids are high, and the antioxidant enzyme activities are relatively low in the brain, so the brain is vulnerable to ischemia/reperfusion. Free radicals may cause oxidative damage to membrane lipids [29]. In the present study, ischemia/reperfusion resulted in elevated level of malondialdehyde (MDA) in brain which were consistent with other studies while treatment with lavender oil greatly reduced the level of MDA. 

To protect against oxidative damage, the brain owns several enzymatic antioxidants, such as superoxide dismutase (SOD), catalase (CAT) and glutathione peroxidase (GSH-Px). SOD catalyzing the conversion of superoxide to hydrogen peroxide. CAT dismutates hydrogen peroxide into water and oxygen. GSH-Px catalyzing the reactions of reduced glutathione [30]. The present investigation showed that the activities of SOD, CAT and GSH-Px decreased significantly in vehicle group compared with the sham-operated group after 2 h of ischemia and 22 h of reperfusion, while the activities of these antioxidant enzymes increased in the mice which were treated with lavender oil.

Lavender oil treatment significantly prevented the rise in carbonyl and MDA levels and up-regulation of SOD, CAT and GSH-Px activities, suggesting that it attenuates the excessive formation of free radicals secondary to reperfusion injury. This is in agreement with the observations that lavender oil possesses antioxidant and anti-inflammatory effects [16]. Glutathione which exists both in reduced and oxidized forms plays an important role in protecting cells against oxidative damage. Reduced glutathione (GSH) is a major endogenous antioxidant in tissues. When cells are exposed to oxidative stress, reduced glutathione becomes glutathione disulfide (GSSG) during the process of binding free radicals. The measurement of GSH/GSSG is useful indicator of oxidative stress and it can be used to monitor the effect of antioxidant [29]. In comparison with the sham-operated group, GSH/GSSG ratio was sharply decreased after 22 h of reperfusion in the vehicle group. That indicated that cerebral ischemia/reperfusion could lessen endogenous antioxidant properties which owing to compensatory increase of GSSG as well as depletion of GSH. Furthermore, GSH/GSSG ratio of the group treated with lavender oil increased significantly when compared with vehicle group.

Mitochondria are the major source of reactive oxygen species (ROS) generated during ischemia/reperfusion [31]. ROS disturb mitochondrial function, inducing the opening of the mitochondrial permeability transition pore and the release of mitochondrial intermembrane proteins to activate apoptotic pathways [32]. Our results showed that ROS production in the brain increased after 22 h of reperfusion. Treatment with lavender oil decreasing ROS generation indicated that the neuroprotection conferred by lavender oil is due to its antioxidative effect of attenuating oxidative stress.

## 3. Experimental

### 3.1. Animals

Adult male Kunming mice, 30–34 g, were housed in a room with temperature of 22–25 °C, relative humidity of 50%–60%, and a 12 h light/12 h dark cycle. All experimental procedures performed were approved by the Institutional Animal Care and Use Committee of National Institute Pharmaceutical Education and Research. 

### 3.2. Chemicals and Reagents

Thedried flowers of lavender (*Lavandula angustifolia* Mill., Lamiaceae) were collected in Yili, Xinjiang, China, in July 2009, and identified by Prof. Ping Yan, School of Life Sciences, Shihezi University. Lavender oil obtained from the dried flowers was used in this study. The oil was obtained via hydrodistillation by using a Clevenger-type apparatus for 4 h and by steam distillation for 5 h. The oils were dried over anhydrous sodium sulphate and stored at 14–16 °C. 1,1,3,3-Tetramethoxypropane was obtained from Fluka Chemical Co. (Ronkonkoma, NY, USA). 2,3,5-Triphenyltetrazolium chloride, hypoxanthine, xanthine oxidase, catalase, 5,5′-dithio-bis(2-nitrobenzoic acid), sodium azide, oxidized glutathione, reduced glutathione and 2′,7′-dichlorodihydrofluorescein diacetate were purchased from Sigma Chemical Co. (St. Louis, MO, USA). All other chemicals and reagents were of analytical grade.

### 3.3. Drug Administration and Surgical Procedure

Mice were randomly divided into sham, vehicle group (maize oil, 10 mL/kg, i.g.), lavender oil (200, 100, 50 mg/kg, i.g.), and edaravone (3 mg/kg, i.p. used as the positive control) treated group. Lavender oil was dissolved in maize oil. Drug or maize oil was administered once a day for three days before ischemia and once at 2 h after the onset of ischemia. After 3 days pretreatment with lavender oil, the operation was performed.

Focal cerebral ischemia/reperfusion (I/R) was induced by occlusion of the middle cerebral artery (MCAO) on the left side according to previously described methods [33] with little modification. Briefly, mice were anesthetized with chloral hydrate (400 mg/kg, i.p.). Body temperature was regulated at 37 °C by homoisothermy bench. Following the skin incision, the left common carotid artery (CCA), the external carotid artery (ECA) and the internal carotid artery (ICA) were carefully exposed and dissected away from adjacent nerves. Microvascular aneurysm clips were applied to the left CCA and the ICA. A coated filament was introduced into an arteriotomy hole, fed distally into the ICA and advanced 12 mm from the carotid bifurcation. The ICA clamp was removed and focal cerebral ischemia started. After ischemia for 2 h, the filament was gently removed. The collar suture at the base of the ECA stump was tightened. The skin was closed, anesthesia discontinued, and the animals were returned to the pre-warmed cages. Mice in the sham group underwent a neck dissection and coagulation of the external carotid artery, but no occlusion of the middle cerebral artery. The data are representative of three independent experiments performed in duplicate.

### 3.4. Measurement of Neurological Deficits

Neurological deficits were determined after 22 h of reperfusion by the method of Longa *et al.* (1989) [34]. Neurological findings were scored as follows: no neurological deficit = 0, failure to extend right paw fully = 1; circling to right = 2, falling to right = 3, did not walk spontaneously and had depressed levels of consciousness = 4. The observer had no knowledge of which treatment had been administered. The above procedures were performed in a blinded manner. Results are means ± S.E.M. of three separate experiments performed in triplicate.

### 3.5. Measurement of Infarct Area

After 22 h of reperfusion, mice were killed by decapitation and the brains were quickly removed and sliced into five 1.5 mm thick coronal sections. The sections were treated with 0.5% 2,3,5-triphenyltetrazolium chloride (TTC) saline solution and incubated at 37 °C for 15 min, followed by 10% formalin fixation overnight according to the method of Kuang *et al*. [35]. Photographs of coronal sections were analyzed by Photoshop 6.0 image analyzer. The infarct areas were expressed as a percentage of the whole area (%).

### 3.6. Measurement of Histopathological Changes

After 22 h of reperfusion, mice were overdosed with anesthetic and perfused with 4% paraformaldehyde in 0.1 M phosphate buffer solution (PBS, pH 7.4). Brains were removed and further fixed in 4% paraformaldehyde at 4 °C for 24 h and then cut into equally spaced blocks. Paraffin-embedded blocks were cut into a series of 5 μm thick slices and stained with hematoxylin-eosin (HE).

### 3.7. Biochemical Measurements

#### 3.7.1. Tissue Preparation

To determine oxidative product contents, antioxidant enzyme activities and redox state, the stored brains were homogenized in 10 volumes of ice-cold sodium chloride in a homogenizer. The homogenate was centrifuged at 4,624 ×g for 15 min. to remove nuclei and cell debris. The supernatant, a suspension of mixed and preserved organelles, was used for the assays.

#### 3.7.2. Measurement of Protein Carbonyl Content

Protein carbonyl content was determined according to the method described by Levine *et al.* [36]. Aliquots of supernatant were incubated with 1% streptomycin sulfate solution for 15 min. The mixture was centrifuged at 3,600 × g for 10 min; the precipitation was incubated with 10 mM dinitrophenylhydrazine (DNPH) in 2 M hydrochloric acid and vortexed every 10 min. The protein was precipitated by adding an equal volume of 20% trichloroacetic acid. After centrifugation at 8,600 × g for 5 min, the pellet was washed three times with ethanol: ethyl acetate (1:1, V/V) to remove excess DNPH. The precipitated protein was redissolved in 6 M guanidine solution and the absorbance of solutions was measured at 370 nm. Carbonyl content was calculated taking the extinction coefficient of 22,000 M^−1^cm^−1^/mg protein. Carbonyl content was expressed as nmol/mg protein.

#### 3.7.3. Measurement of Malondialdehyde (MDA) Content

MDA content in brain homogenate was determined using the thiobarbituric acid (TBA) method [37] with modification. In brief, aliquots of 10% supernatant were incubated with 0.8% TBA. The mixture was heated in 95 °C water bath for 1 h. Afterwards, *n*-butanol and pyridine (15:1, V/V) was added and the mixture was centrifuged. The organic phase was collected to measure fluorescence at excitation and emission wavelengths of 515 and 553 nm, respectively. 1,1,3,3-Tetramethoxypropane, which is converted to MDA, was used as standard. MDA content was expressed as nmol/mg protein.

#### 3.7.4. Measurement of Superoxide Dismutase (SOD) Activity

Superoxide dismutase (SOD) activity was measured according to a previously described procedure with minor modification [38]. Briefly, 18 μL of different concentration supernatant was incubated with 18 μL solution A (1 mM hydroxylamine/1 mM hypoxanthine) and 18 μL solution B(10 mU/mL xanthine oxidase with 0.05 mM EDTA-Na_2_) and 36 μL distilled water added to the 96-pore plate were vortexed and then left standing in a 37 °C water bath for 30 min. One hundred and eighty μL solution C (5.2 mM sulfanilic acid/77.2 μM *N*-1-naphthylethylenediamine in 16% acetic acid) was then added to the final solution and the mixture was allowed to stand at 30 °C for 15 min. The absorbance of the test solution was measured at 550 nm. One unit of SOD activity was defined as the amount that shows 50% inhibition. SOD activity was expressed as U/mg protein.

#### 3.7.5. Measurement of Catalase (CAT) Activity

Catalase (CAT) activity was measured according to method described previously [39]. Briefly, 20 μL of supernatant was incubated with 100 μL substrate (65 mM hydrogen peroxide in 60 mM phosphate buffer solution, pH = 7.4) at 37 °C for 1 min. The enzymatic reaction was then stopped with 100 μL ammonium molybdate (32.4 mM). The absorbance of the test solution was measured at 405 nm. One unit of CAT activity was defined as the amount of CAT required to decompose 1 μmol/L of hydrogen peroxide in min. CAT activity was expressed as U/mg protein.

#### 3.7.6. Measurement of Glutathione Peroxidase (GSH-Px) Activity

Glutathione peroxidase (GSH-Px) activity was measured according to a previously described procedure with minor modification) [40]. Briefly, 5 μL of supernatant was mixed with 10 μL each of ethylenediamine tetraacetic acid (EDTA), sodium azide, GSH, hydrogen peroxide and 20 μL buffer. The reaction mixture was incubated at 37 °C for 10 min followed by the addition of 10% trichloroacetic acid (TCA). After centrifugation at 1,400 g for 10 min, the supernatant was collected and mixed with 150 μL disodium hydrogen phosphate and 50 μL 5,5′-dithio-bis(2-nitrobenzoic acid) (DTNB). The absorbance of the test solution was measured at 412 nm. One unit of GSH-Px activity was defined as the GSH-Px in 1 mg protein that led to the decrease of 1 μM GSH in the reactive system per minute. GSH-Px activity was expressed as U/mg protein.

#### 3.7.7. Measurement of Protein Content

Protein content was measured by the method of Bradford [41] using bovine serum albumin (BSA) as standard protein.

#### 3.7.8. Measurement of GSH/GSSG Ratio

Oxidative stress was assessed through measurement of the GSH/GSSG ratio [42]. The concentrations of total glutathione (T-GSH), reduced glutathione (GSH) and oxidized disulfide (GSSG) were measured by an enzymatic method. Briefly, T-GSH was assayed using the 5,5-dithio- bis(2-nitrobenzoic) acid (DTNB)-GSSG reductase recycling. GSSG was measured by measuring 5-thio-2-nitrobenzoic acid (TNB) which was produced from the reaction of reduced GSH with DTNB. The rate of TNB formation was measured at 412 nm. The concentration of reduced GSH was obtained by subtracting GSSG from T-GSH.

#### 3.7.9. Measurement of Reactive Oxygen Species (ROS) in Brain Mitochondria

Mice were sacrificed by decapitation. Brain mitochondria isolation was conducted as previously described. The brain was quickly removed and placed in beakers containing ice-cold isolation buffer (0.25 M sucrose containing 10 mM Tris-HCl, pH = 7.4, 1 mM EDTA, and 250 µg/mL BSA). The brain tissue was repeatedly washed with the isolation buffer to remove adhering blood and 10% (W/V) homogenate was prepared in a homogenizer. The nuclei and cell debris were precipitated by centrifugation at 4,624 g for 10 min and discarded. The supernatant was subjected to a further centrifugation at 15,413 g for 15 min. Finally the mitochondria were suspended in the above buffer at a volume of 20–25 mg/mL. All procedures were performed at 4 °C.

ROS production in mouse brain mitochondria was monitored by using the membrane-permeable fluorescent probe 2′,7′-dichlorofluorescin diacetate (DCFH-DA) [43]. Mitochondria isolated from different groups (0.5 mg/mL) were incubated with 10 µM DCFH-DA at 37 °C for 1 h, and the fluorescence intensity of DCF was measured at an excitation wavelength of 488 nm and emission wavelength of 525 nm. ROS level was expressed as percentage in fluorescence relative to vehicle group. All results are means ± S.E.M. of three separate experiments performed in triplicate.

#### 3.7.10. Statistical Analysis

All quantitative data were analyzed with SPSS 10.0 software and expressed as mean ± S.E. Statistical comparisons were performed with Student’s *t*-test. Differences were considered significant at *P* <0.05.

## 4. Conclusions

Lavender oil offered neuroprotection in focal cerebral ischemia/reperfusion. Neuroprotection shown by lavender oil may be attributed to inhibition of protein oxidation, lipid peroxidation, augmentation in endogenous antioxidant defense and reduction in mitochondria-generated ROS. Our study suggests that lavender oil might have a potential as a therapeutic agent for the prevention of cerebral ischemic damage.
